# Cardiac Resynchronization Therapy: Reconsidering Its Essence as a Treatment for Electrical Dyssynchrony in Heart Failure

**DOI:** 10.1002/joa3.70268

**Published:** 2026-01-09

**Authors:** Michio Ogano, Yu‐ki Iwasaki, Jun Tanabe, Wataru Shimizu, Kuniya Asai

**Affiliations:** ^1^ Department of Cardiovascular Medicine Shizuoka Medical Center Shimizu Japan; ^2^ Department of Cardiovascular Medicine Nippon Medical School Bunkyo Tokyo Japan

**Keywords:** cardiac resynchronization therapy, electrical dyssynchrony, heart failure, left ventricular lead positioning, QRS duration

## Abstract

Cardiac resynchronization therapy (CRT) essentially targets electrical dyssynchrony, one of the key drivers in heart failure (HF). Its efficacy depends on both the quality (QRS morphology) and quantity (QRS duration) of dyssynchrony, requiring individualized patient selection. While drug therapy for HF has a limited effect on this substrate, early implantation of CRT can prevent irreversible remodeling and facilitate optimization of medical therapy. CRT should be recognized as an essential component within a comprehensive strategy for HF management in patients with appropriate indications.

## Graphical abstract

Flow of cardiac resynchronization therapy (CRT) in heart failure management. Heart failure (HF) patients with electrical dyssynchrony are first identified through screening. If electrical dyssynchrony contributes to HF deterioration, CRT is initiated to improve electrical synchrony. CRT elevates the “cardiac synchrony” component, leading to stabilization of the HF condition. When HF subsequently worsens, the electrocardiogram (ECG) is evaluated. If the ECG shows no change compared with the post‐CRT pattern of improved electrical synchrony, interventions targeting other drivers of HF are performed. If the ECG demonstrates changes indicating loss of the previously improved electrical synchrony, CRT reprogramming or multi‐site pacing strategy should be undertaken.

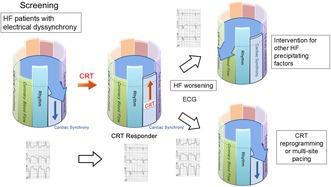

## 1 Introduction

Cardiac resynchronization therapy (CRT) is an established treatment for patients with symptomatic chronic heart failure (HF). Its efficacy has been demonstrated in numerous large‐scale randomized clinical trials [1, 2, 3], including landmark studies such as COMPANION [4] and MADIT‐CRT [2], which firmly established CRT as a cornerstone therapy for selected HF patients. However, approximately 30% of patients fail to achieve the expected clinical benefit, which remains a topic of debate [5]. This review reconsiders the fundamental principles of CRT and discusses the unresolved challenges associated with its use.

## 2 Assessment of the Effects of HF Treatment

### 2.1 What Exactly Does CRT Improve in Patients With HF?

In previous clinical trials, the definition of a “CRT responder” has varied, encompassing endpoints such as all‐cause mortality, hospitalization for HF, degree of left ventricular reverse remodeling, and improvement in exercise tolerance [6]. HF is a multifactorial and progressive disease; thus, when implementing therapy, it is essential to identify which of the many drivers is primarily being targeted.

**FIGURE 1:**
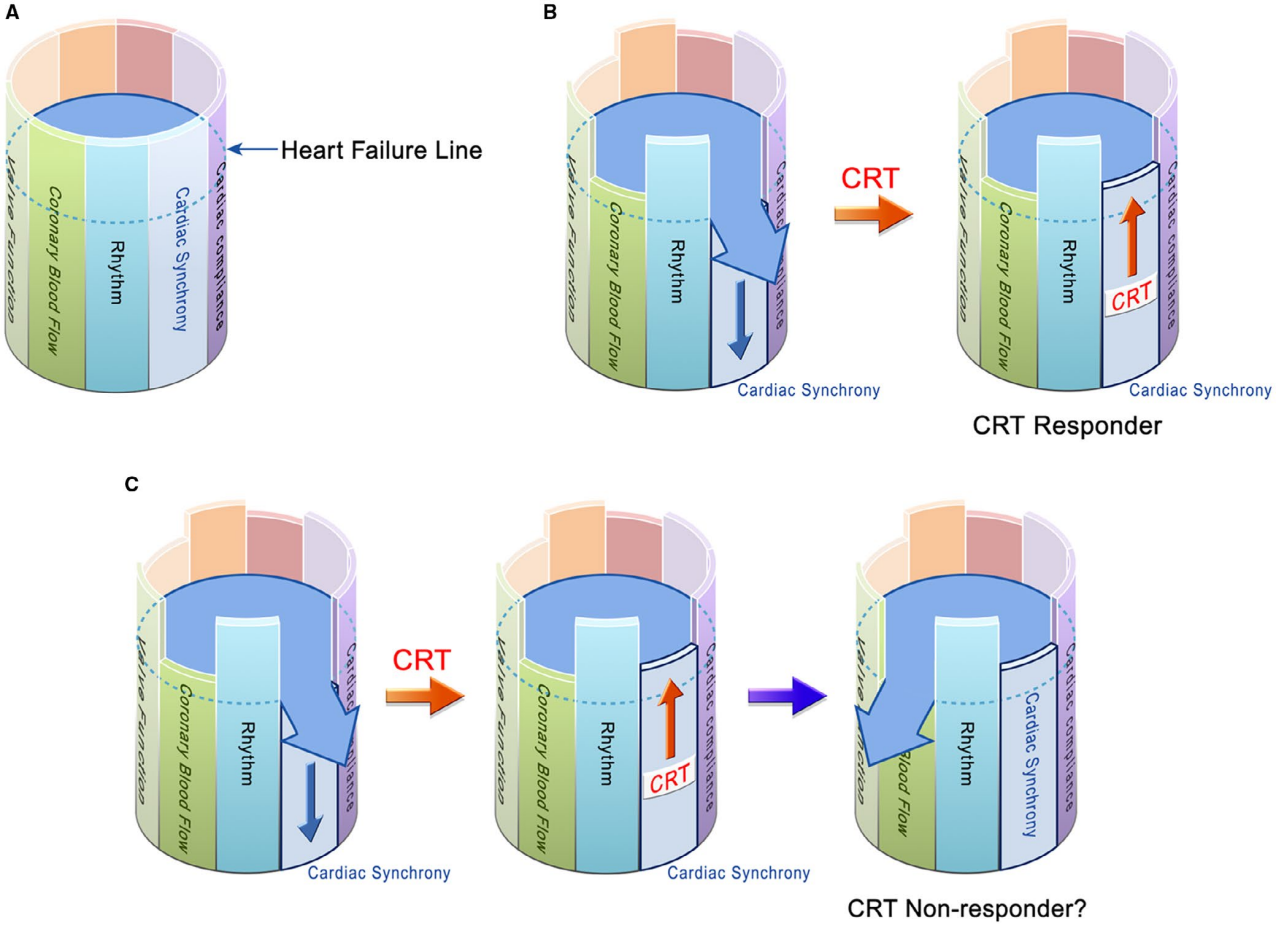
Multifactorial model of heart failure. (A) The barrel is filled with water, and each stave represents an individual function that determines cardiac performance. The “heart failure line” is set at a certain level. (B) For example, when a new left bundle branch block develops, cardiac synchrony is impaired. This lowers the corresponding stave, causing the water level to exceed the heart failure line and leak out. The leakage represents a heart failure event. In this case, introducing cardiac resynchronization therapy (CRT) elevates the stave of synchrony, preventing further leakage and thereby stabilizing heart failure. No new heart failure events would be expected for a while, and this case is considered a CRT responder. (C) However, heart failure is a multifactorial disease. It is uncommon for all other determinants of cardiac function to remain intact when heart failure occurs due to impaired synchrony. For instance, some patients may have concomitant reductions in coronary blood flow. Although CRT can restore synchrony and stabilize heart failure, it does not improve coronary stenosis, and thus the stave for coronary blood flow remains low. During the subsequent course, heart failure may recur due to progression of coronary artery disease. In such a scenario, although a heart failure event occurs after CRT, which may traditionally be classified as a CRT non‐responder, CRT has in fact restored synchrony and should be regarded as having provided a therapeutic response.

Figure 1A illustrates the multifactorial model of HF. In this analogy, a barrel is filled with water, and each stave represents a factor determining cardiac function. For example, if new‐onset left bundle branch block (LBBB) occurs, cardiac synchrony is impaired, and one stave lowers (Figure 1B). Once that stave drops to the point where water begins to leak, acute HF is assumed to occur. In this scenario, CRT can restore cardiac synchrony, raise the stave, and stop the leakage, thereby treating acute HF. Moreover, CRT can maintain cardiac synchrony thereafter, preventing new episodes of acute decompensation. Such a patient would be defined as a CRT responder. However, in real‐world practice, it is rare for acute HF triggered by loss of cardiac synchrony to occur in complete isolation from other contributing factors. For instance, as in Figure 1C, a patient presenting with acute HF due to loss of cardiac synchrony may also have reduced coronary perfusion, lowering another stave of the barrel. Even if CRT successfully restores synchrony and resolves acute decompensation, progressive coronary artery disease during follow‐up may impair perfusion enough to trigger another episode of acute HF. In such a case, the patient would be classified as a CRT nonresponder according to conventional definitions, despite the fact that CRT effectively corrected the targeted cardiac dyssynchrony. Thus, when defining the effect of HF therapy, it is essential to identify which driver is being addressed. Even if negative endpoints—such as death, recurrent HF hospitalization, cardiovascular events, insufficient reverse remodeling, or limited improvement in exercise capacity—are observed after treatment, these do not necessarily indicate that the therapy was ineffective or that the patient is a nonresponder.

## 3 The Essence of CRT and How to Evaluate CRT Response

### 3.1 Which Driver in HF Does CRT Actually Address?

As its name suggests, CRT is a therapy aimed at restoring cardiac “resynchronization,” meaning it treats loss of synchrony within the heart. Cardiac dyssynchrony can be categorized into electrical dyssynchrony, as assessed by electrocardiography (ECG), and mechanical dyssynchrony, as assessed by wall motion imaging modalities such as echocardiography. Because CRT is fundamentally an electrical therapy delivered via cardiac pacing, its direct target is electrical dyssynchrony. Mechanical dyssynchrony is closely related but improves indirectly once electrical activation is restored.

PROSPECT study [7] has shown that mechanical dyssynchrony indices do not provide clinical benefit for selecting CRT candidates and vary markedly with loading conditions. This preload dependency has been demonstrated experimentally [8] and was reaffirmed in the ECHO‐CRT trial [9]. Our hemodialysis observations likewise showed marked shifts in mechanical dyssynchrony between pre‐ and post‐dialysis, despite unchanged ECG findings (Figure S1, Videos S1 and S2). In contrast, electrical dyssynchrony is the upstream driver of ventricular activation, and improvement in electrical parameters—such as QRS narrowing—strongly predicts CRT response [10, 11]. Accordingly, current guidelines [12] prioritize electrical dyssynchrony for CRT indication, whereas mechanical dyssynchrony parameters are not recommended for CRT indication (Class III).

**FIGURE 2:**
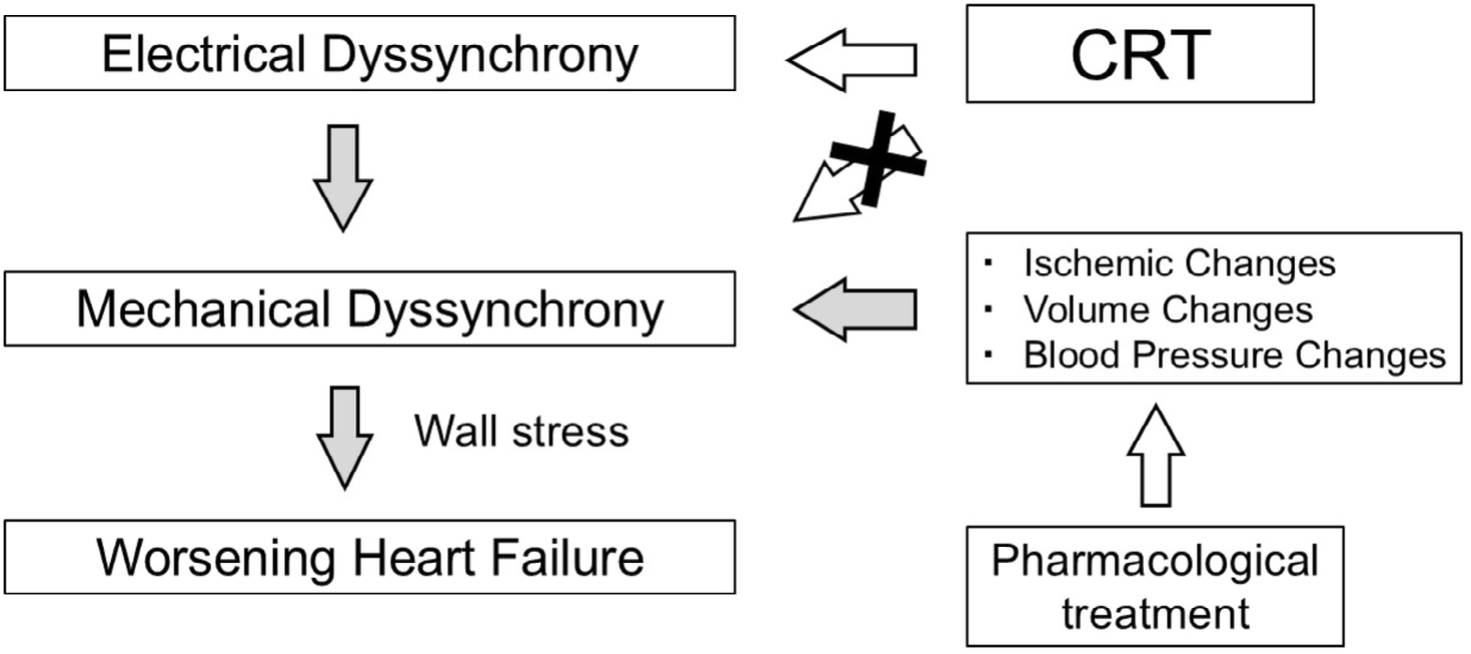
Diagram of the relationship between electrical and mechanical dyssynchrony. CRT is not a therapy that directly acts on mechanical dyssynchrony, but rather corrects mechanical dyssynchrony through the modulation of electrical dyssynchrony. The direct target of CRT is electrical dyssynchrony. Electrical dyssynchrony represents an upstream factor contributing to worsening heart failure. Gray arrows indicate pathological pathways, whereas open arrows indicate therapeutic interventions. CRT, cardiac resynchronization therapy.

Mechanical dyssynchrony can alter intracardiac pressure gradients, impose wall stress on the myocardium, and contribute to HF progression [13]. Persistent mechanical dyssynchrony is associated with worse HF prognosis [14]. However, as noted above [8], multiple factors—blood pressure, effective circulating plasma volume, and wall motion abnormalities due to coronary ischemia, among others—can cause such changes. Electrical dyssynchrony resulting from conduction system disorders is distinct in nature from these other factors. Electrical dyssynchrony is upstream of mechanical dyssynchrony as a driver in HF. Because CRT is fundamentally a pacing‐based electrical intervention, its direct therapeutic target is electrical dyssynchrony, while improvements in mechanical dyssynchrony are indirect (Figure 2).

Placement of the left ventricular (LV) lead is critical for improving electrical dyssynchrony [12]. Since the site of latest electrical activation often coincides with the site of latest mechanical contraction [15], imaging modalities such as echocardiography or MRI can be used to guide LV lead placement [16, 17]. However, because the essence of CRT is correction of electrical dyssynchrony, the optimal target is the electrically latest activated site (Q‐LV site) [12]. When cardiac MRI demonstrates that the electrically latest activated segment is occupied by dense scar, however, LV lead placement at that site is not recommended; instead, the lead should be positioned in the latest‐activated viable segment. Late gadolinium‐enhancement cardiac MRI is particularly useful for delineating scar, and pacing within scarred myocardium has been associated with attenuated reverse remodeling and worse clinical outcomes after CRT [18].

Previous reports [19] have shown that placing the LV lead where Q‐LV > 95 ms yields better outcomes after CRT. A prolonged Q‐LV interval indicates that the region is substantially delayed in activation, making it the most pathophysiologically appropriate site for achieving effective resynchronization. Moreover, the degree of QRS narrowing after CRT correlates with prognosis, making it a useful measure of post‐implant efficacy [10]. Techniques such as fusion‐optimized CRT—which utilizes the patient's intrinsic atrioventricular conduction and adjusts the AV interval to achieve the narrowest possible QRS—have been associated with improved outcomes after CRT [20].

Conduction system pacing (CSP), which has recently gained considerable attention, can often achieve greater improvement in electrical synchrony than conventional biventricular pacing because it recruits the heart's physiological conduction pathways. As a result, CSP frequently produces narrower QRS complexes, and several randomized trials [21, 22] have demonstrated better clinical improvement with CSP compared with conventional CRT. Large multicenter randomized trials are currently underway, and further evidence is anticipated. Despite these promising findings, it is essential to emphasize that CSP—like conventional biventricular CRT—is fundamentally a therapy directed at correcting electrical dyssynchrony. Even when CSP successfully restores electrical synchrony, it does not address all upstream drivers of heart failure. Therefore, whether using CSP or conventional CRT, therapy should be implemented within a comprehensive, multifactorial HF management strategy rather than viewed as a standalone solution.

**FIGURE 3:**
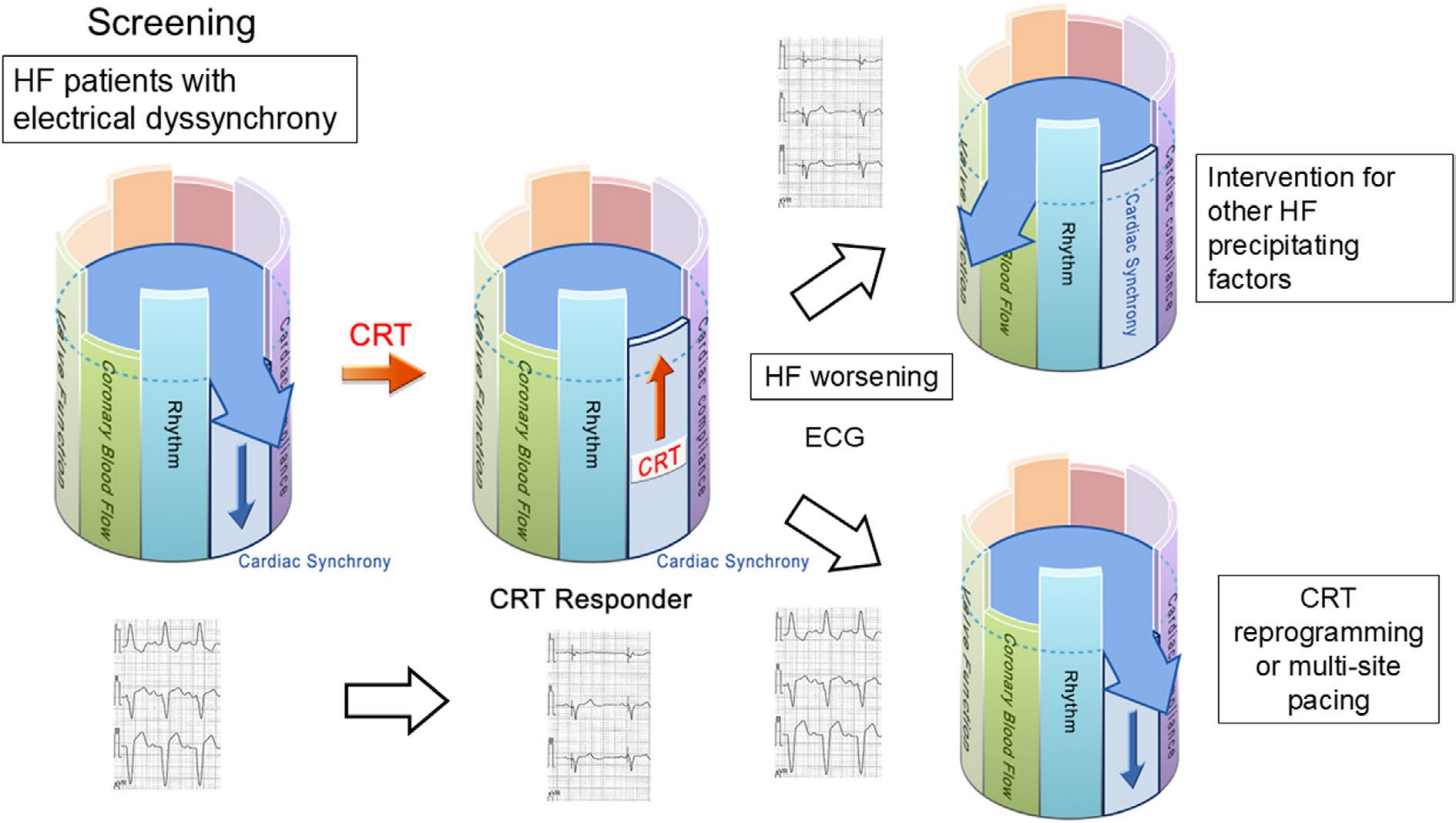
Flow of cardiac resynchronization therapy (CRT) in heart failure management.

In many studies, CRT efficacy has been assessed at 6 or 12 months post‐implant using outcomes such as all‐cause mortality and HF re‐hospitalization. However, CRT is a therapy that corrects one specific driver—electrical dyssynchrony—among many in HF. Even in cases where electrical synchrony is improved after CRT implantation, patients may experience heart failure exacerbation without reverse remodeling. In such situations, it is important to carefully assess for other potentially correctable drivers of heart failure, and the device should not be stopped without a careful reassessment [23]. Conversely, if post‐CRT improvement in electrical dyssynchrony is insufficient or worsens, device re‐programming or alternative strategies [24, 25] such as multi‐site or multi‐point pacing (as described later) [26] should be considered (Figure 3).

Because CRT is a therapy that targets electrical dyssynchrony, assessment of CRT response should fundamentally be based on post‐implant ECG evaluation. Specifically, improvement in electrical synchrony should be confirmed by analyzing QRS duration, QRS axis, and A–V/V–V timing as markers of effective resynchronization. Patients in whom these electrical indices fail to improve—or even worsen—despite appropriate lead position and programming can be regarded as true electrical nonresponders.

## 4 Quantity and Quality of Electrical Dyssynchrony

Electrical dyssynchrony due to conduction system disorders can be understood in terms of both quality (QRS morphology) and quantity (QRS duration). The quality of electrical dyssynchrony refers to the QRS morphology on ECG, which can be broadly classified into left bundle branch block (LBBB), right bundle branch block (RBBB), and nonspecific intraventricular conduction delay (IVCD). The quantity of electrical dyssynchrony is represented by QRS duration.

Large‐scale clinical trials [1, 2, 3, 4] have shown that CRT is most effective in electrical dyssynchrony of high quality (LBBB morphology) and high quantity, defined as wide QRS (> 150 ms). In other words, patients with LBBB and wide QRS are most likely to benefit from CRT, and current guidelines [27] strongly recommend CRT for this population. It is intuitive that CRT is effective in wide QRS, as the greater the quantity of correctable electrical dyssynchrony, the greater the potential benefit. But why is CRT particularly effective in LBBB compared with other QRS morphologies?

In LBBB, the degree of hemodynamic stress imposed on the heart is greater than with other qualities of electrical dyssynchrony [28, 29]. In LBBB, electrical activation of the LV lateral wall is delayed because conduction must detour via the right bundle to the apex, then spread to the LV, so that the LV lateral wall is activated substantially after the septum. As a result, by the time the LV lateral wall begins to contract, the septum has already completed contraction. This creates a strong pressure gradient from the lateral wall toward the septum.

**FIGURE 4:**
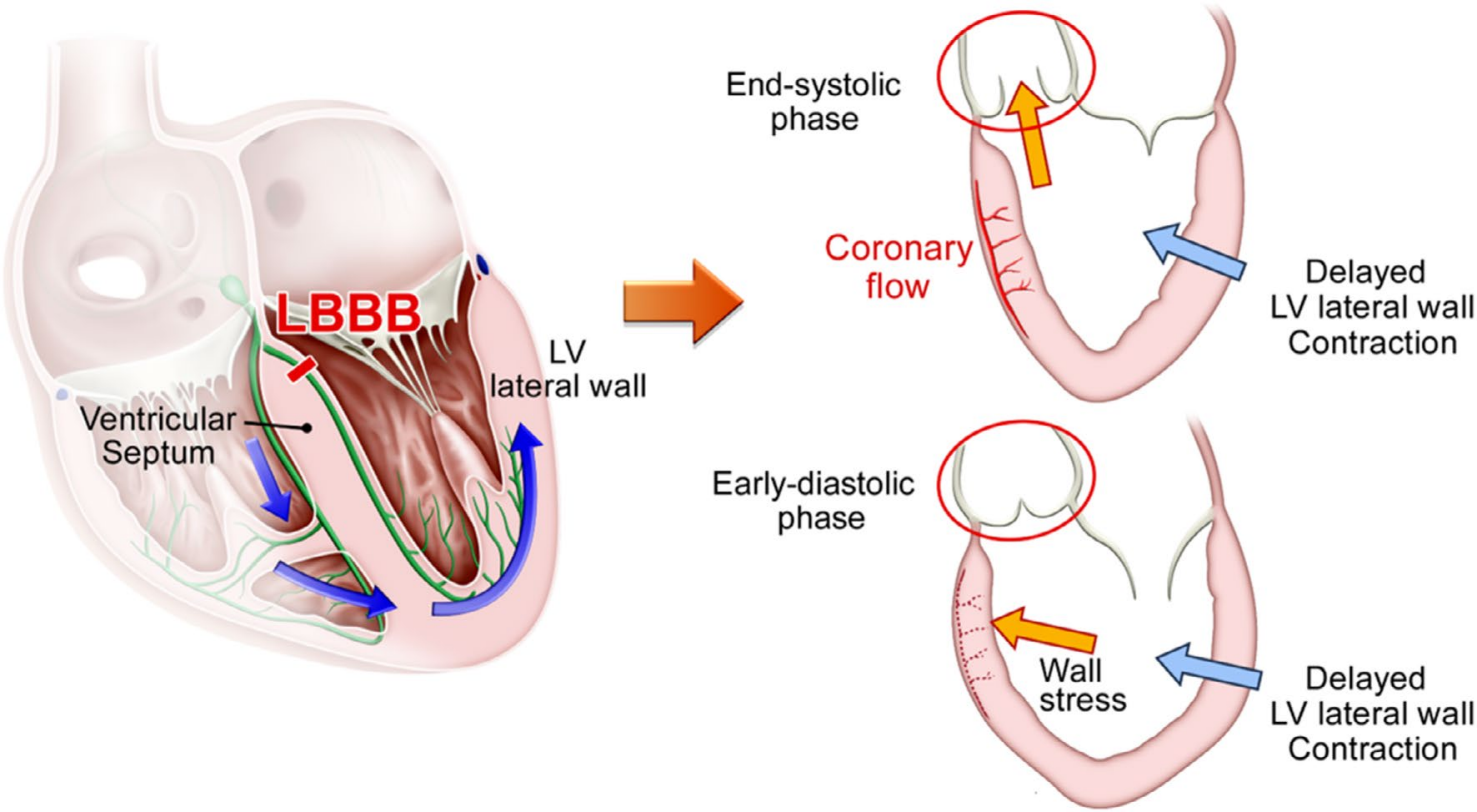
Mechanism of interventricular septal ischemia in left bundle branch block. In left bundle branch block (LBBB), electrical conduction to the left ventricular (LV) lateral wall proceeds through the right bundle branch and detours within the myocardial layer from the apex, resulting in delayed activation and contraction of the LV lateral wall compared with the ventricular septum. This delayed contraction directs LV intracavitary pressure toward the septum and imposes significant wall stress. When this septal pressure shift occurs at end‐systole, the aortic valve remains open and LV pressure is partially relieved into the aorta. However, when it occurs in early diastole, the aortic valve is closed and the increased intracavitary pressure can impair coronary blood flow in the septal region.

Ono et al. [28] studied changes in myocardial pressure within the interventricular septum in normal hearts and in those with experimentally induced LBBB. In the normal group, peak intramyocardial pressure in the septal region occurred during systole, whereas in LBBB, the peak shifted to early diastole. Coronary blood flow is determined by the pressure gradient between the aortic sinus of Valsalva and the myocardium; this gradient depends on a rapid fall in myocardial pressure in early diastole. If septal myocardial pressure rises during early diastole, the gradient decreases, leading to relative ischemia [30] (Figure 4). Because right ventricular systolic pressure is much lower than left ventricular pressure, delayed right ventricular contraction in RBBB produces far less wall stress than delayed LV lateral wall activation in LBBB.

We have previously shown, using myocardial scintigraphy, that patients with nonischemic cardiomyopathy and LBBB exhibit ischemia in the septal region, and that CRT can improve this ischemia [29]. For wall stress high enough to induce ischemia in the septum, the LV lateral wall must be sufficiently delayed. Even if the lateral wall contracts late, if the pressure on the septum occurs in late systole when the aortic valve is open, LV pressure can be vented into the aorta. Ischemia occurs when wall stress on the septum is applied in early diastole, when the aortic valve is closed. Among the various qualities of electrical dyssynchrony, LBBB produces the most delayed activation of the LV lateral wall, and the degree of delay is quantified as QRS duration. Therefore, LBBB with wide QRS represents the combination of quality and quantity most likely to generate septal wall stress, and CRT can most effectively reduce this hemodynamic burden, resulting in the greatest clinical benefit.

When predicting CRT efficacy, which should be prioritized—quality or quantity? In previous studies [31] comparing four groups—LBBB + wide QRS (> 150 ms), non‐LBBB + wide QRS (> 150 ms), LBBB + moderately wide QRS (120–150 ms), and non‐LBBB + moderately wide QRS (120–150 ms)—patients with LBBB + moderately wide QRS had better outcomes after CRT implantation than those with non‐LBBB + wide QRS. This suggests that, in predicting CRT benefit, quality of electrical dyssynchrony should be prioritized over quantity. We categorized the features of electrical dyssynchrony suitable for CRT from the perspectives of quality and quantity and summarized them in a table. This table allows a reasonable prediction of the expected CRT response. The rationale for each type of electrical dyssynchrony will be described in detail in the following sections.

## 5 Individualized Approaches to Electrical Dyssynchrony

**FIGURE 5:**
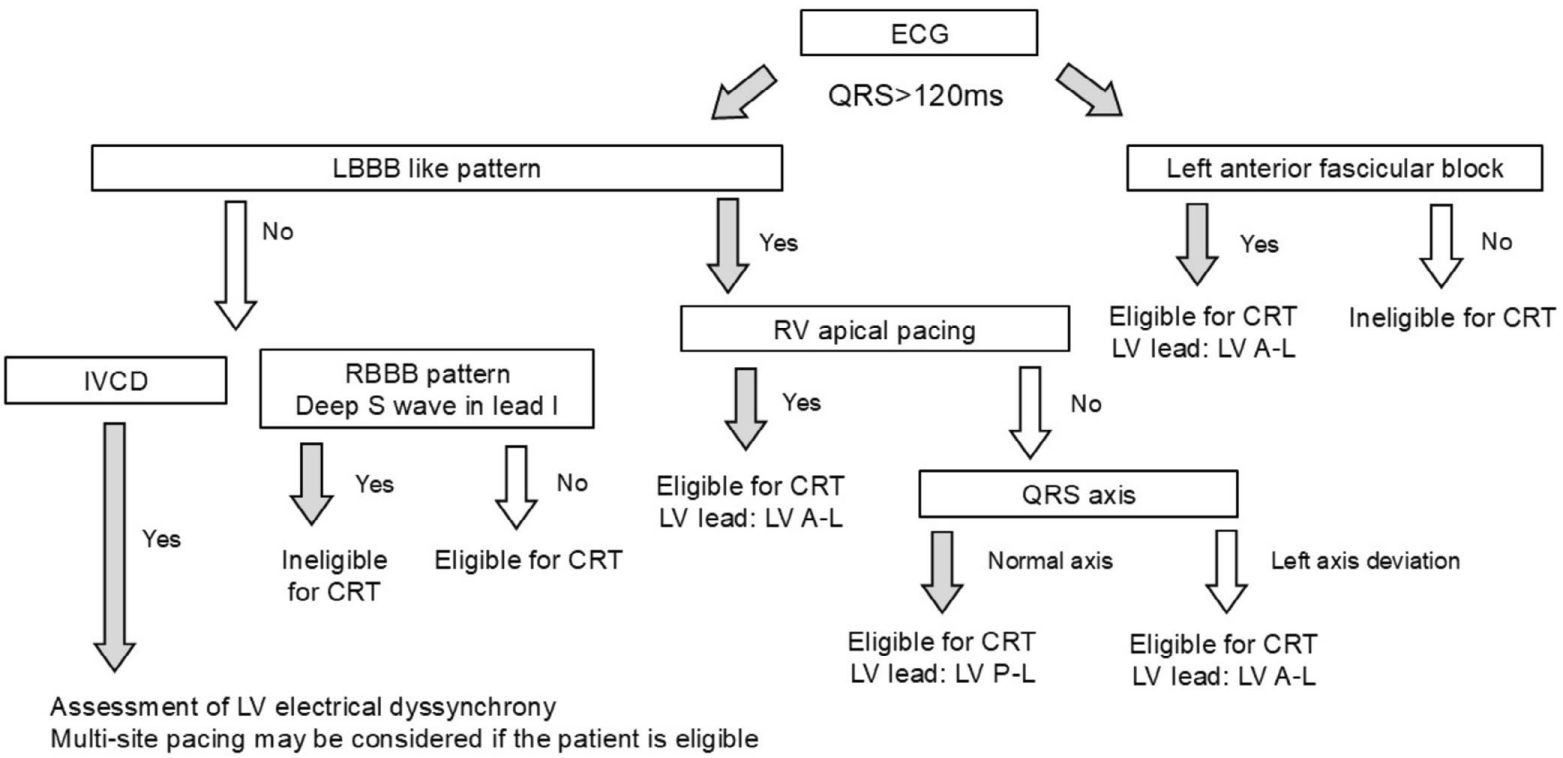
Flowchart for CRT eligibility and recommended LV lead positioning. This flowchart summarizes an ECG‐based approach for evaluating eligibility for cardiac resynchronization therapy (CRT) and determining the recommended left ventricular (LV) lead position. Patients with QRS ≥ 120 ms are categorized according to QRS morphology, axis, and pacing status. “LBBB‐like patterns” include typical and atypical LBBB morphologies, as well as RV apical pacing patterns that resemble LBBB with left axis deviation. For eligible patients, the recommended LV lead position is indicated: LBBB, left bundle branch block; RBBB, right bundle branch block; LV A–L, left ventricular anterolateral region; LV P–L, left ventricular posterolateral region.

It is clinically meaningful to estimate the expected benefit of CRT before implantation. The therapeutic effect of CRT varies according to the quality and quantity of each type of electrical dyssynchrony (Table 1). In the following sections, we describe practical tips for clinical decision‐making when considering CRT implantation for each electrical dyssynchrony quality pattern, including pathophysiological considerations and recommended left ventricular lead positions. We have also summarized, in a flowchart, how to evaluate clinical eligibility and determine the recommended LV lead placement (Figure 5).

**TABLE 1:** Qualitative and quantitative features of electrical dyssynchrony and their association with the expected response to cardiac resynchronization therapy.

	ED quality	ED quantity		Expected CRT effect
LBBB	+++	150 ms <	++	+++++
		120–150 ms	+	++++
RV pacing	+++	150 ms <	++	+++++
		120–150 ms	+	++++
RBBB	+ (if S wave (−) in lead I)	150 ms <	++	+++
		120–150 ms	+	++
IVCD	+ (if LV activation delay)	150 ms <	++	+++
		120‐150 ms	+	++
LAHB	+	120‐150 ms	+	++

Abbreviations: CRT, cardiac resynchronization therapy; ED, electrical dyssynchrony; IVCD, intraventricular conduction disturbance; LAHB, left anterior hemi‐block; LBBB, left bundle branch block; RBBB, right bundle branch block; RV right ventricle.

### 5.1 Typical CRT Indications: Left Bundle Branch Block (LBBB)

In LBBB, the pathophysiologic mechanism described above produces a marked disparity in left ventricular (LV) pressure gradients (wall stress), leading to substantial clinical improvement after CRT in most cases. However, ECG‐based definitions of LBBB vary among reports [32], and individual patient presentations can differ widely. Upadhyay et al. [33] have identified the exact site of conduction block in patients with an ECG diagnosis of LBBB by placing an electrode catheter in the LV septum. They found that 38% of patients, conduction from the His bundle to the Purkinje fibers was intact, and the block site was located distal to the Purkinje fibers. Such cases, although showing LBBB morphology on ECG, should be classified electrophysiologically as intraventricular conduction delay. In these patients, conduction is diffusely slowed within the LV myocardium, making identification of the site of latest activation difficult and limiting the potential for CRT to improve electrical dyssynchrony.

Among patients with LBBB, some show a normal frontal plane QRS axis, while others present with left axis deviation (right axis deviation is very rare in clinical practice). Patients with left axis deviation have significantly more delayed LV conduction than those with a normal axis [34], and outcomes after CRT implantation are worse in this group [35, 36]. One possible explanation is that severe underlying heart disease (e.g., myocardial infarction scar) contributes to the axis deviation, with prognosis reflecting the severity of the underlying condition. Another possibility is related to LV lead placement. Sciarra et al. [37] have reported that in LBBB with a normal axis, the site of maximal Q‐LV is located in the LV lateral wall, whereas in LBBB with left axis deviation, the site of maximal Q‐LV is in the LV anterior wall. In most large trials [1, 2, 3, 4] demonstrating CRT efficacy, LV leads were placed in the lateral or posterolateral wall, and many subsequent studies have followed this practice without targeting the actual site of maximal delay. Thus, in patients with LBBB and left axis deviation, routine placement of the LV lead in the lateral or posterolateral wall may fail to capture the site of maximal Q‐LV, potentially worsening prognosis.

### 5.2 LBBB‐Like (LBBB‐Equivalent) Conduction Patterns: Pacing‐Induced Dyssynchrony/Upgrade to CRT

Right ventricular (RV) pacing creates nonphysiological intraventricular conduction and induces pacing‐related cardiomyopathy in approximately 12% of patients [38]. RV apical pacing produces an electrical dyssynchrony pattern similar to LBBB and is usually associated with a wide QRS. CRT has been shown to be effective when implanted de novo in patients anticipated to require chronic pacing [39], and also when used as an upgrade in patients who have developed LV dysfunction as a result of pacing dependency [40].

Extending our previous work on septal ischemia in LBBB, we investigated nonischemic cardiomyopathy patients dependent on RV apical pacing [41]. Myocardial scintigraphy revealed a reduction in tracer uptake in the septal region similar to that seen in LBBB, and the degree of reduction correlated with paced QRS duration. Upgrading to CRT improved the reduced uptake in these patients.

**FIGURE 6:**
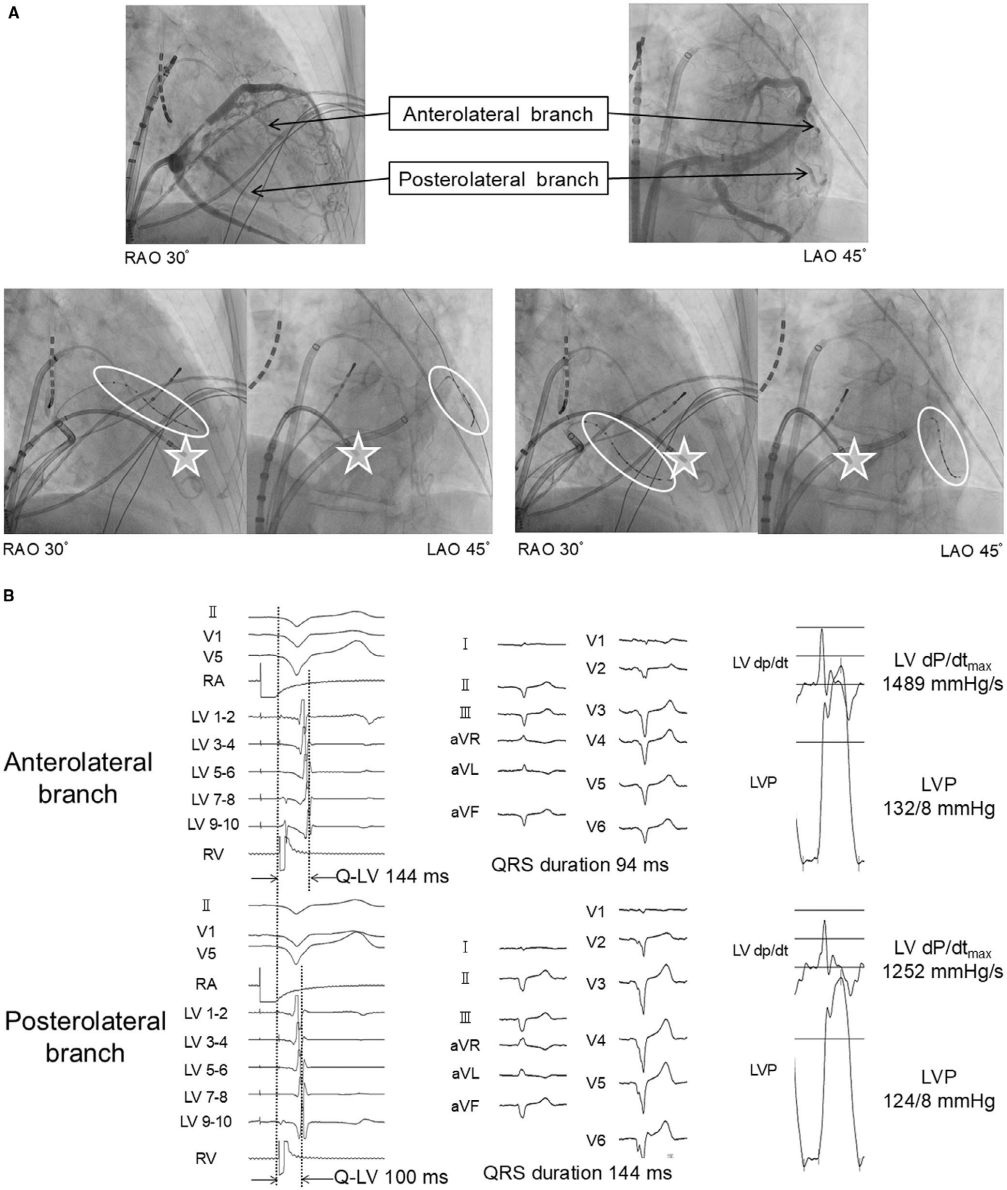
Site of maximal intraventricular conduction delay during right ventricular pacing and the effects of biventricular pacing. (A) Coronary sinus angiography was performed in an 80‐year‐old male. The anterolateral and posterolateral branches are depicted (upper panel). Electrode catheter was inserted into each branch (white circle). During RV apical stimulation (white star), Q‐LV was measured at each branch (lower panel). (B) The Q‐LV interval was longer at the anterolateral site than at the posterolateral site (left panel). Simultaneous biventricular pacing showed that the QRS duration was shorter at the anterolateral site than at the posterolateral site (middle panel). The LV dP/dt_max and LVP were higher at the anterolateral site than at the posterolateral site (right panel). The anterolateral site showed a longer Q‐LV interval, a greater QRS narrowing with simultaneous biventricular pacing, and a higher LV dP/dt_max, indicating that it is a desirable location for left ventricular lead placement. Reproduced with permission from Ogano et al. [42].

LV lead position is also important in upgrades from RV apical pacing. Although RV apical pacing produces an electrical dyssynchrony pattern similar to LBBB, it is not identical. As noted in the LBBB section, patients with LBBB may present with either a normal axis or left axis deviation, whereas RV apical pacing produces an ECG pattern similar to that of LBBB with left axis deviation. In this pattern, the site of latest LV activation is typically the LV anterolateral wall, not the LV posterolateral wall. We studied Q‐LV timing during RV apical pacing and found it was longer in the LV anterolateral wall than in the LV posterolateral wall [42]. Furthermore, simultaneous pacing from the LV anterolateral wall during RV apical pacing shortened QRS duration and improved hemodynamics compared with LV posterolateral pacing (Figure 6). Therefore, in patients undergoing CRT upgrade from RV apical pacing, LV lead placement in the LV anterolateral region may be reasonable based on these observations.

CRT upgrade for pacing‐induced cardiomyopathy due to RV apical pacing can reliably improve electrical dyssynchrony and often produces substantial clinical benefit, but some studies have shown limited improvement. In the RAFT trial [3], which included upgrade cases, a subgroup analysis failed to show clear benefit in the upgrade population. One possible reason was that LV leads were placed in the LV posterolateral wall, rather than in the optimal LV anterolateral site for this patient group. No studies have yet defined the optimal LV site for latest activation in pacing‐induced cardiomyopathy caused by non‐apical RV pacing.

### 5.3 Atypical Morphologies: Right Bundle Branch Block (RBBB) and Intraventricular Conduction Disturbance (IVCD)

In patients with RBBB, the site of latest intraventricular activation is typically located in the right ventricle (RV), and there is generally no delayed activation site within the LV. If there is no LV conduction delay, placing an LV lead and pacing will not correct any existing electrical dyssynchrony, making such an approach clinically unhelpful.

When conduction delay is present in the RV, the quality of electrical dyssynchrony differs from that in the LV; the latest activated site may be in the RV free wall or the RV outflow tract. Moreover, because RV pressures are lower than LV pressures, the magnitude of wall stress caused by mechanical dyssynchrony in association with electrical delay is also lower in the RV than in the LV. Therefore, even if a lead is positioned to target the latest‐activated site within the RV, the degree of clinical improvement is likely to be less than in LBBB. (An exception is in pediatric patients, where CRT with RV lead placement has been reported to be effective for electrical dyssynchrony associated with right‐sided HF [43].)

**FIGURE 7:**
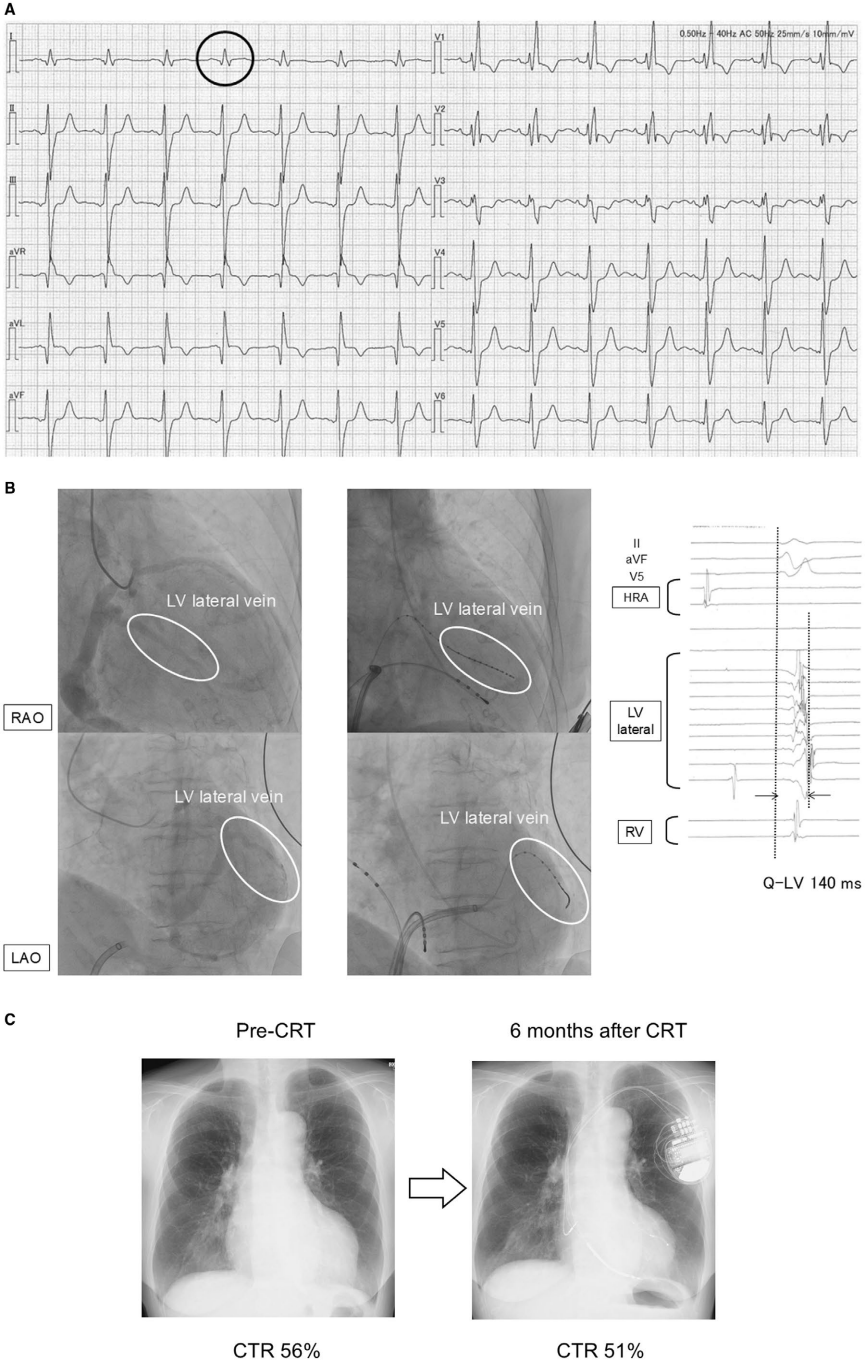
A case of successful cardiac resynchronization therapy for bundle branch delay. (A) A woman in her 70s. Pre‐CRT electrocardiogram showing right bundle branch block with a very small S wave in lead I (black circle), consistent with bilateral bundle‐branch delay. (B) Coronary venography demonstrated the left ventricular (LV) lateral vein (left panel). An electrode catheter was advanced to this site (middle panel), and Q‐LV measurement showed a prolonged interval of 140 ms (right panel). HRA = high right atrium. (C) The LV lead was implanted at the LV lateral wall and cardiac resynchronization therapy (CRT) was performed. Left ventricular ejection fraction improved from 34% to 45%, and the cardiothoracic ratio on chest radiograph decreased from 56% to 51%.

CRT is effective in RBBB patients only when there is concomitant electrical dyssynchrony within the LV—that is, when RBBB is accompanied by LV conduction delay. Auricchio et al. [44] created three‐dimensional activation maps of both ventricles in RBBB patients and reported that some of them had delayed conduction in the LV lateral wall. Tzogias et al. [45] have examined ECGs from patients who developed iatrogenic RBBB due to mechanical compression during ablation or device procedures. Among patients with pre‐existing LBBB, some did not develop complete AV block when iatrogenic RBBB was induced, a group termed bilateral bundle branch delay. The hallmark ECG finding in such patients was the absence of the S wave in lead I. In contrast, patients with normal baseline conduction, left anterior fascicular block, or left posterior fascicular block showed a deep S wave in lead I after iatrogenic RBBB. This loss of the S wave in lead I is considered a specific marker of RBBB with concomitant LV conduction delay. The same finding has been confirmed in RBBB patients with LV lateral wall conduction delay documented by three‐dimensional mapping [44]. Pastore et al. [46] have shown that RBBB patients with good CRT outcomes often have “atypical” RBBB patterns on ECG, characterized by absent or markedly diminished S waves in lead I, further supporting the notion that CRT is effective in RBBB patients with concomitant LV conduction delay (Figure 7). When considering CRT in RBBB patients, it is therefore important to evaluate for the presence of concomitant LV electrical dyssynchrony, and the absence of an S wave in lead I can be a useful indicator.

However, it should be noted that many RBBB patients with reduced LV ejection fraction have severe underlying heart disease and multiple HF drivers. Even if CRT improves electrical dyssynchrony, HF may still worsen due to progression of other drivers, and reverse remodeling may be insufficient. Under conventional definitions, such cases are prone to being labeled as CRT nonresponders.

Patients who do not have LBBB or RBBB but exhibit prolonged QRS duration on ECG are classified as having intraventricular conduction disturbance (IVCD). This category encompasses a heterogeneous group of cases. As with RBBB, for CRT to be effective, there must be electrical dyssynchrony within the LV, and pre‐procedural assessment is essential.

In the ENHANCE‐CRT study [47], non‐LBBB patients (35% of whom had IVCD) were evaluated using both anatomical criteria and Q‐LV measurements to predict CRT benefit, but no significant difference in clinical improvement was found between the two approaches. However, despite the fact that 55.6% of enrolled patients had a baseline QRS duration ≥ 150 ms, the average Q‐LV times measured in this study were only 81.07 ms in the anterior interventricular vein and 94.22 ms in the LV anterolateral branch—both below 95 ms. This finding suggests that, even among patients with wide QRS, there was relatively little correctable LV electrical dyssynchrony in the study population.

Conversely, Ploux et al. [48] have analyzed myocardial scintigraphy patterns in IVCD and found that patients whose conduction disturbance resembled LBBB morphology did benefit from CRT, underscoring again the importance of the quality of electrical dyssynchrony.

Similar to RBBB, many IVCD patients have conduction delay within the LV—especially distal to the Purkinje network—due to severe underlying heart disease. Consequently, mortality and HF re‐hospitalization rates due to drivers other than electrical dyssynchrony are often high. Therefore, during follow‐up after CRT, it is important not to hastily judge such patients as nonresponders without considering the contribution of other drivers.

### 5.4 Narrower QRS but Meaningful Electrical Dyssynchrony; Left Fascicular Block

Electrical dyssynchrony arises from conduction system disease, and left fascicular block can also cause electrical dyssynchrony. Compared with LBBB, however, the hemodynamic burden imposed by electrical dyssynchrony in left fascicular block is generally less severe, and isolated left fascicular block is unlikely to precipitate HF on its own.

**FIGURE 8:**
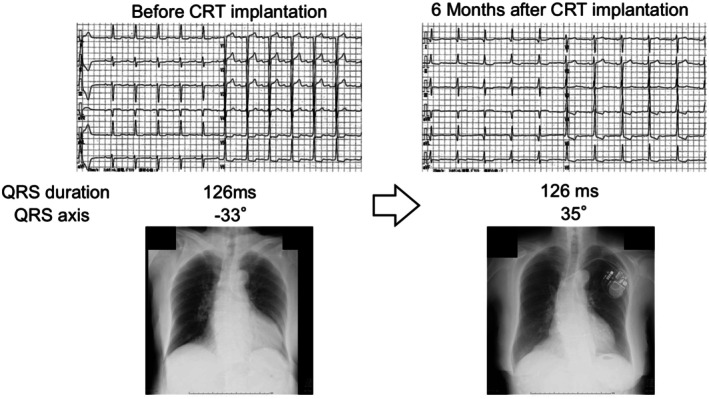
A case of successful cardiac resynchronization therapy for left anterior fascicular block. An 82‐year‐old woman with a baseline electrocardiogram showing a QRS duration of 126 ms and left anterior fascicular block. A left ventricular (LV) lead was implanted at the anterolateral site with delayed conduction, and cardiac resynchronization therapy (CRT) was initiated. After CRT, the QRS duration remained 126 ms, but the QRS axis shifted from −33° to 35°, indicating normalization. At the 6‐month follow‐up, chest radiography demonstrated improvement in the cardiothoracic ratio, and the patient's New York Heart Association (NYHA) functional class improved from III to I. Reproduced with permission from Ogano et al. [50].

Nonetheless, patients with left fascicular block have been shown to have worse prognosis than those without [49], suggesting that there is room for therapeutic intervention. We have reported a case [50] in which the site of latest LV activation was identified in a patient with left anterior fascicular block, and an LV lead was placed at that site to deliver fusion pacing with the patient's intrinsic right bundle conduction. This strategy restored electrical synchrony and produced marked clinical improvement (Figure 8). Of note, because the left anterior fascicle supplies the LV anterolateral papillary muscle, the mitral regurgitation thought to be caused by the fascicular block improved after CRT implantation.

Although QRS prolongation in left fascicular block is usually mild—meaning the quantity of electrical dyssynchrony is limited—its quality can still generate significant intraventricular pressure gradients within the LV, making it a potentially responsive substrate for CRT. This again illustrates that, when considering electrical dyssynchrony, quality should be prioritized over quantity.

Recent study [51] on left bundle branch pacing has reported that patients who show left anterior or posterior fascicular block after pacing have worse prognosis than those without such block. This suggests that residual left fascicular block may represent uncorrected electrical dyssynchrony that imposes hemodynamic burden. In patients at risk for HF exacerbation due to such residual block, consideration should be given to additional strategies such as LOT‐CRT, discussed below.

### 5.5 Advanced Techniques: Multi‐Point Pacing Including HOT‐CRT and LOT‐CRT

Previous studies have reported that with conventional CRT—pacing from one RV site and one LV site—approximately 30% of patients still have residual cardiac dyssynchrony after implantation [52]. The basic concept of multisite pacing is to increase the number of pacing sites to correct this residual dyssynchrony.

The TRIP Study [53] evaluated the efficacy of triple‐site pacing (one RV site + two LV sites in different coronary sinus branches) in patients with chronic atrial fibrillation. In this crossover trial, triple‐site pacing resulted in significantly greater reverse remodeling compared with conventional two‐site pacing. Similarly, the TRUST Study [54] established a triple‐site pacing system in patients with sinus rhythm and showed, in interim results, the superiority of triple‐site over two‐site pacing. On the other hand, the V3 Study [55], which added a second LV lead to patients deemed nonresponders to standard CRT (converting them to triple‐site pacing), did not demonstrate significant clinical improvement.

In CRT candidates such as those with LBBB, where the quality of electrical dyssynchrony imposes a substantial hemodynamic burden, conventional two‐site pacing (one RV + one LV) already corrects the quality component effectively. Any remaining dyssynchrony in such cases is quantitative, and the potential for additional clinical benefit from correcting residual quantity is limited.

In an experimental study, Ploux et al. [56] created LBBB in mice and tracked the latest activation site while sequentially increasing the number of pacing sites. They found that cardiac electrical synchrony and hemodynamics improved markedly when increasing from one to two pacing sites, but improvements beyond two sites were minimal. In our own work [57], we placed temporary pacing leads directly in coronary sinus branches before CRT implantation and compared QRS duration and LV dP/dt changes between conventional two‐site pacing and triple‐site pacing (one RV + two LV). Hemodynamic improvement with triple‐site pacing, compared with two‐site pacing, was observed in only 29.3% of patients. Based on these temporary pacing results, we implanted CRT systems with either two‐site or triple‐site pacing and followed patients for 7 years. Mortality rates did not differ significantly between the groups, but triple‐site pacing was associated with better prognosis in terms of CCS class and HF hospitalization rates.

Recently, in conduction system pacing (CSP), additional LV leads placed in coronary sinus branches have been used to perform hybrid strategies such as His‐optimized CRT (HOT‐CRT) [58] and left bundle branch area pacing–optimized CRT (LOT‐CRT) [59] for residual electrical dyssynchrony. However, in most cases, CSP alone already corrects the quality of electrical dyssynchrony sufficiently, and as with triple‐site pacing, the number of patients who truly require HOT‐CRT or LOT‐CRT is small. Rave et al. [60] investigated the applicability of LOT‐CRT based on electrocardiographic parameters in 19 patients and found that only one patient met the criteria. Similarly, Tam et al. [61] employed a multi‐electrode vest and the CardioInsight system to evaluate the pacing configuration that provided the greatest improvement in electrical dyssynchrony among 20 patients. Their results showed that multisite pacing achieved the most favorable reduction in electrical dyssynchrony in only a small number of patients, specifically two with HOT‐CRT and one with LOT‐CRT. Artificial pacing also carries the risk of creating new electrical dyssynchrony. Because LV pacing from the epicardial surface has different transmural conduction times and directions compared with endocardial‐to‐epicardial activation [62, 63, 64], placing an LV lead away from the latest activation site can paradoxically create new dyssynchrony [65]. Parale et al. [66] reported that in patients with baseline QRS < 135 ms after left bundle branch pacing, LOT‐CRT often widened the QRS, indicating induction of new dyssynchrony by additional LV pacing. Recently, Jastrzębski et al. [67] reported that LOT‐CRT should be considered only in patients with baseline QRS ≥ 150–171 ms and post–left bundle branch pacing QRS ≥ 135 ms.

## 6 Timing of CRT Implementation

Electrical dyssynchrony is a chronic driver in HF that imposes sustained hemodynamic burden. In particular, LBBB produces a high‐quality form of electrical dyssynchrony that exerts substantial stress on the heart, making early consideration of CRT important. However, current guidelines [12] recommend CRT implantation only after a three‐month period of optimized medical therapy (OMT).

The rationale for this three‐month OMT period originates from earlier large‐scale CRT trials, in which enrolled patients had already been on guideline‐directed therapy. Nevertheless, the fact that CRT was effective in OMT‐treated patients does not necessarily mean it would be ineffective in those not yet on OMT; rather, the effect of CRT in OMT‐naïve patients remains unknown [68]. Moreover, the definition of “optimized” medical therapy is not clearly established. In prior CRT trials [69], β‐blocker use at the time of CRT implantation was about 80%, ACE inhibitor/ARB use about 70%, and MRA use about 50%. In the more recent Adapt CRT study [70], only 6% of patients were on β‐blockers, ARNI, and MRA at the time of implantation. Electrical dyssynchrony cannot be corrected pharmacologically, and the effect of guideline‐directed drug therapy on this specific driver is limited [71, 72]. Mignone et al. [73] have shown that CRT improves prognosis independently of OMT. The number needed to treat (NNT) for CRT to prevent all‐cause mortality is reported to be 24 at 1 year and 8 at 3 years—figures comparable to—and in no way inferior to—those achieved with each of the well‐established drug therapies recommended in the guidelines for symptomatic chronic HF [74].

The 2021 ESC guidelines [75] state that, in patients with LBBB, drug therapy has no direct effect on conduction abnormalities causing electrical dyssynchrony and that most drug trials (e.g., ARNI, SGLT2 inhibitors, ivabradine) enrolled very few CRT‐eligible patients. While these observations suggest that CRT does not necessarily need to be delayed for full optimization of medical therapy, it is important to acknowledge that robust randomized evidence supporting “early CRT” is lacking. Accordingly, the ESC guidance uses intentionally cautious language—CRT may be considered rather than definitively recommended—and further trials in OMT‐naïve or partially treated populations are needed to clarify the optimal timing.

Currently, CRT remains underutilized [76], and many eligible patients are missed. Delayed implantation allows irreversible remodeling to progress, diminishing CRT efficacy [68]. CRT response is particularly poor among patients with advanced chronic HF who are dependent on catecholamines [77]. Even in patients for whom OMT is difficult to achieve, early CRT can stabilize hemodynamics, facilitate subsequent titration of OMT [78, 79], and reduce or discontinue unnecessary diuretics, leading to improved prognosis [80]. Leyva et al. [81] reported that in CRT‐eligible patients, implantation should occur at least between the first hospitalization and any subsequent re‐admission.

## 7 Conclusion

CRT is a therapy that corrects electrical dyssynchrony—one of the drivers for HF. Both the quality and quantity of electrical dyssynchrony differ among patients, and tailored intervention is required for each case. Heart failure events after CRT implantation do not necessarily indicate that the patient is a nonresponder; rather, the potential contribution of other HF drivers should be carefully evaluated before making such a judgment. Earlier implementation of CRT can facilitate titration of guideline‐directed medical therapy resulting in improved prognosis. CRT should be recognized as an important component within a comprehensive strategy for HF management.

## Funding

The authors have nothing to report.

## Conflicts of Interest

Dr. Ogano has received consulting fees and honoraria from Medtronic. The other authors declare no conflicts of interest.

## Supporting information


**Figure S1:** Changes in mechanical dyssynchrony associated with alterations in intravascular plasma volume. Speckle‐tracking echocardiography performed before and after hemodialysis in a patient on maintenance dialysis with a QRS duration of 130 msec and an ejection fraction of 30% (Videos S1 and S2: Notably, the electrocardiograms attached in the lower left panels demonstrate no changes in QRS duration, axis, or heart rate before and after dialysis. However, marked changes in wall motion are observed). Mechanical dyssynchrony varies between pre‐ and post‐dialysis, with improvement in dyssynchrony observed after dialysis compared with before. SD_6_ = standard deviations among 6 areas.

**Video S1:** Short‐axis transthoracic echocardiographic views obtained before and after hemodialysis.

**Video S2:** Speckle‐tracking echocardiographic analysis corresponding to Video S1.
