# Supplementary figures and images for: Cardiac Resynchronization Therapy: Reconsidering Its Essence as a Treatment for Electrical Dyssynchrony in Heart Failure
Source: J Arrhythm. 2026 Jan 9;42(1):e70268. doi: 10.1002/joa3.70268 (PMC12789663; doi:10.1002/joa3.70268)

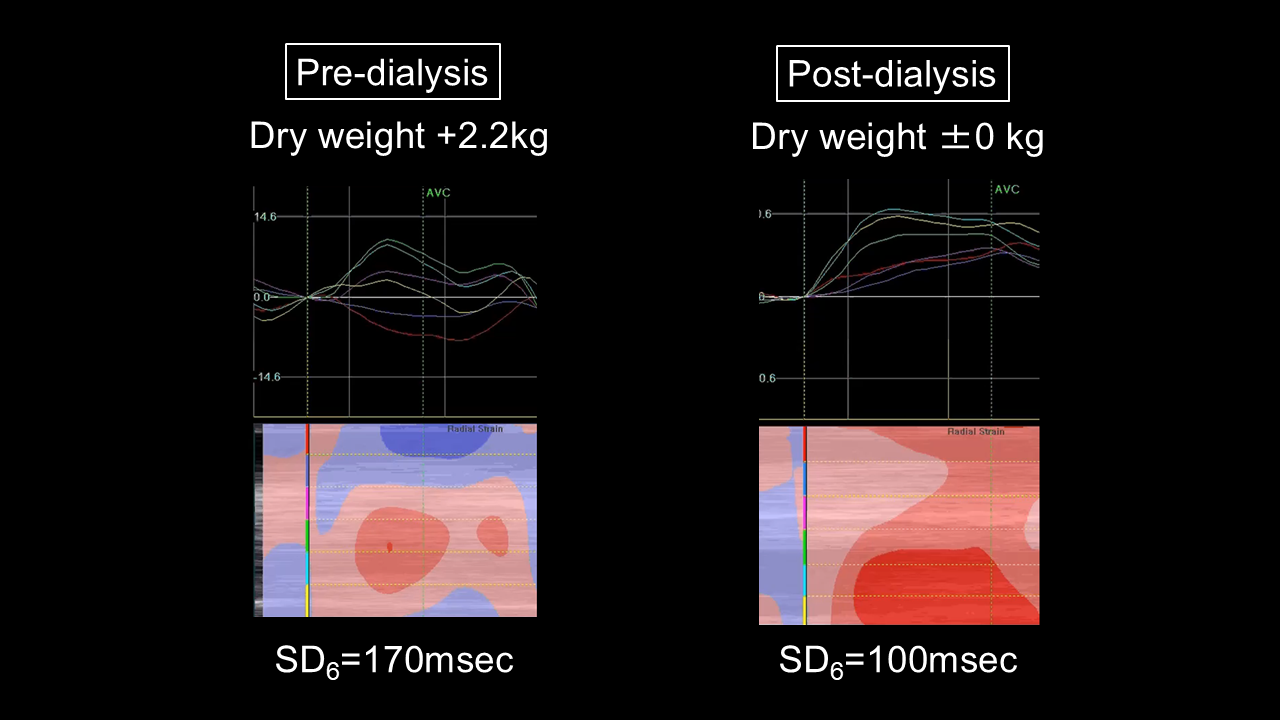

Supplement: Supplementary file 1 — Figure S1: Changes in mechanical dyssynchrony associated with alterations in intravascular plasma volume. Speckle‐tracking echocardiography performed before and after hemodialysis in a patient on maintenance dialysis with a QRS duration of 130 msec and an ejection fraction of 30% (Videos S1 and S2: Notably, the electrocardiograms attached in the lower left panels demonstrate no changes in QRS duration, axis, or heart rate before and after dialysis. However, marked changes in wall motion are observed). Mechanical dyssynchrony varies between pre‐ and post‐dialysis, with improvement in dyssynchrony observed after dialysis compared with before. SD6 = standard deviations among 6 areas. [file JOA3-42-e70268-s002.tif]
